# Preschool children in out-of-hours primary care – a questionnaire-based cross-sectional study of factors related to the medical relevance of health problems

**DOI:** 10.1186/s12875-017-0702-5

**Published:** 2017-12-28

**Authors:** Grete Moth, Linda Huibers, Astrid Ovesen, Morten Bondo Christensen, Peter Vedsted

**Affiliations:** 1 Research Unit for General Practice, Department of Public Health, Aarhus University, Bartholins Alle 2, 8000 Aarhus, Denmark; 2 Section for General Medical Practice, Department of Public Health, Aarhus University, Bartholins Alle 2, 8000 Aarhus, Denmark

**Keywords:** Denmark, General practice, Out-of-hours, Appropriateness, Severity, Reason for encounter

## Abstract

**Background:**

Out-of-hours primary care (OOH-PC) is intended to provide medical care services for health problems that cannot wait until normal office hours. Children under five years of age represent about 19% of all OOH-PC contacts in Denmark, and the frequency of calls assessed as *severe* by health professionals is markedly lower for children than for other age groups. Several studies have questioned the appropriateness of the parents’ use of OOH-PC. We aimed to identify factors associated with calls from parents of pre-school children concerning perceived non-severe health problems that were ranked by the triaging GPs as more appropriate for GP office hours (defined as ‘medically irrelevant’).

**Methods:**

We used data from a cross-sectional study performed in the Central Denmark Region for a 1-year period during 2010–2011. GPs in the OOH-PC assessed random contacts, and a questionnaire was subsequently sent to registered patients. Associations between different factors and the medical irrelevance of contacts were estimated with a generalised linear model to calculate the prevalence ratio (PR).

**Results:**

Among all included 522 telephone consultations and 1226 face-to-face consultations, we identified 71 (13.6%) telephone consultations and 95 (7.8%) face-to-face consultations that were both assessed as *non-severe* by the parents and *more appropriate for GP office hours* by the GPs. For telephone consultations, contacts at other times than 4–8 pm on weekdays were statistically significantly associated with medical irrelevance. Additionally, symptoms of longer duration than 24 h were statistically significantly associated medical irrelevance.

**Conclusions:**

A large part of the calls to the Danish OOH-PC concern children. The results indicate that some of these calls are made for other than strictly medical reasons. To achieve more effective use of available resources, it might seem relevant to aim at directing more contacts directly to daytime care. However, future studies to enhance our knowledge on parents’ motivation and behaviour would be recommendable.

## Background

Out-of-hours primary care (OOH-PC) in Denmark is organised in large cooperatives consisting of general practitioners (GPs) as in many other western countries [1–3]. The OOH-PC is intended for urgent medical problems that cannot wait until normal office hours. Nevertheless, several studies have shown that 50–77% of calls are assessed as *inappropriate* or *non-urgent* by health professionals and could have been directed to a GP in daytime or managed as self-care [4–6]. Consequently, the workload at the OOH-PC is high, and the demands are significant [7].

Studies across countries have shown that young children account for 5–19% of the OOH-PC contacts [8–10]. The proportion of calls assessed as severe is lower for children than for other age group [11]. According to a Danish study, 46% of parents who contacted OOH-PC did not consider their child’s condition to be truly serious or urgent [12]. Furthermore, a Dutch study showed that about half of patients thought that OOH-PC was intended for all health-related problems, including non-urgent issues [4].

Therefore, it is relevant to analyse the medical relevance of contacts concerning children as this could provide an understanding of the OOH-PC use for this group. Our aim was to examine contacts for 0–5-year-old children at the OOH-PC regarding reasons for encounter (RFE), symptom duration, time of contact, urbanisation and severity of health problem as assessed by parents and the OOH-PC GP on duty. Furthermore, for contacts considered as non-severe by parents, we explored factors that were associated with being assessed as more suitable for own GP within office hours by the triaging GPs in order to gain knowledge on how to identify the potentially avoidable contacts.

## Methods

### Design

We conducted a cross-sectional study based on secondary analyses of data from a survey on RFE and disease patterns in the Danish OOH-PC (the LV-KOS cohort study) in the Central Denmark Region [8]. During the 1-year study period (1 June 2010 ─ 31 May 2011), data on OOH-PC contacts were generated on the basis of GP registrations of contacts and patient questionnaires. A total of 385 of all 700 GPs in the region participated in the survey at least once (55.0%), and a GP collected data for 96% of all telephone calls in each shift. In total, 21,457 random contacts were registered: 7810 telephone calls, 6973 face-to-face consultations and 6674 home visits.

### Setting

The OOH-PC provides care for the 1.25 million inhabitants in the region and registers 670,000 calls annually. In the Danish OOH-PC services, patients must call the OOH-PC call centres, which are managed by fully licensed GPs. Nearly 60% of calls are terminated as telephone consultations (medical advice or direct admission to the hospital) and 40% as telephone referrals (referral to subsequent face-to-face consultation (clinic visit or home visit)).

### Data collection

At the start of each shift at the OOH-PC, one GP from each type of consultation (phone, clinic, home visit) could sign up electronically in the study as they logged on to the computerized patient administration system. Participating GPs received an electronic pop-up questionnaire after terminating every tenth telephone call, every third clinic consultation and every home visit. The questions focused on a range of characteristics of the contact, such as the RFE (stated in text), and duration of symptoms. Moreover, a question concerned whether the patient ought to have attended own GP within office hours (either before or after the OOH-PC contact) instead of contacting the OOH-PC.

Two to three days after the registered OOH-PC contact, the patients received a postal questionnaire, including a question about their assessment of the severity of the health problem prompting a call. Patients registered with more than one contact in the study received only one questionnaire (after the first contact). Moreover, contacting patients with permanent address outside the region did not receive a questionnaire. Data on age, date, time and type of contact were collected from the electronic patient administration system*.*

### Data management

Focusing on children aged 0–5 years and their parents’ decision to call the OOH-PC, we categorised the children into two age groups: <3 years and 3–5 years. Contacts were categorised into two groups: telephone consultations and face-to-face consultations (clinic visits, home visits).

RFEs stated in text in the patient records were obtained and manually coded using the International Classification of Primary Care, 2nd Edition (ICPC-2) [13] by a specifically trained medical student, who was supervised by the researchers. The four most frequently applied RFE diagnoses were identified, and all additional RFEs (143 in total) formed a group of “other RFEs”. Duration of symptoms was categorised into four groups: <5 h, 5–12 h, >12–24 h and >24 h. Based on the contact pattern in OOH-PC, which showed a peak during the first opening hours, the time of contact was categorised into three groups: weekday evenings (4–8 pm), weekday nights (8 pm-8 am), and weekends and holidays. Geographical location was based on the postcode of the patient’s home address and categorised according to number of citizens in the postcode area: rural (≤5000) and urban (>5000).

The patient-assessed severity was dichotomised into “potentially severe” (combining “S*evere and life-threatening”* and *“Severe but not life-threatening”*) and “not severe” (combining “*Not severe, but I needed to talk to a doctor”* and *“Not ill, but I had some questions”*).

### Analysis

We compared respondents and non-respondents among the parents on the basis of relevant variables available in this study (age groups, type of contact, symptom duration, geographical location and GP-assessed medical relevance for the OOH-PC). Baseline characteristics of the included contacts were presented for telephone consultations and face-to-face consultations together with the proportion of contacts that the participating OOH-PC GPs considered to be more appropriate for the daytime GP. Next, we identified the subgroup of calls that the OOH-PC GPs considered more appropriate for the daytime GP and that were also assessed as non-severe by the parents. This subgroup is henceforth referred to as “medically irrelevant contacts” in this paper. In a generalised linear model (GLM), we examined, for each consultation type, the association between being categorised in this subgroup and age, duration of symptoms, time of contact and geographical location; these associations were presented as estimates of prevalence ratios (PR) and 95% confidence intervals (95% CI). In this analysis, answers of “don’t know” to assessment of severity were excluded as were RFEs with too few observations. Analyses were performed by Stata 14 statistical software.

## Results

During the study period, 3857 contacts regarding children aged 0–5 years were registered. Of these, 292 were excluded, primarily because they were registered with more than one contact. Of the remaining 2923 contacts, completed patient questionnaires were returned for 1748 contacts (59.8%), which were included in the analyses (522 telephone consultations and 1226 face-to-face consultations). Respondents and non-respondents did not differ significantly, except for the GP-assessment of appropriateness. The GPs found that 18% of the contacts from respondents compared with 24% from non-respondents could have been more appropriately targeted to the daytime GP than to the OOH-PC (*P* < 0.001) (Table 1).

**Table 1:** Comparison of characteristics between respondents and non-respondents

		Respondents (*n*=) (%)	Non-respondents (*n*=) (%)	*P*-value
Age	0–2 years	1216 (69.6)	829 (70.5)	0.568
	3–5 years	532 (30.4)	346 (29.5)	
Type of contact	Telephone consultation	522 (29.9)	357 (30.4)	0.764
	Face-to-face consultation	1.226 (70.1)	818 (69.6)	
Symptom duration	<5 h	439 (25.1)	278 (23.7)	0.069
	5–12 h	396 (22.7)	271 (23.1)	
	12–24 h	376 (21.5)	233 (19.8)	
	>24 h	517 (29.6)	365 (31.1)	
	Missing	20 (1.1)	28 (2.4)	
Geographical location	Urban	727 (41.6)	524 (44.6)	0.220
	Rural (<5000 inhab. Per zip code)	923 52.8)	582 (49.5)	
	Missing	98 (5.6)	69 (5.9)	
More appropriate for the daytime GP according to the OOH-PC GP	No	1.309 (74.9)	803 (68.3)	<0.001
	Yes	317 (18.1)	284 (24.1)	
	Don’t know	122 (7.0)	88 (7.5)	

More contacts concerned children aged 0–2 years than children aged 3–5 years; this was found for both types of consultations (Table 2). The duration of symptoms was most often under five hours for telephone consultations (31.8%) and over 24 h for face-to-face consultations (31.6%). In total, 60.7% of telephone consultations and 40.6% of face-to-face consultations were assessed as non-severe by the parents.

**Table 2:** Characteristics of OOH-PC contacts for preschool children and proportions considered by GPs to be more appropriately targeted to the daytime GP (*N* = 1748)

		Telephone consultations		Face-to-face contacts	
		*n* (%)	Appropriate for daytime GP (%)	*n* (%)	Appropriate for daytime GP (%)
All calls		522 (100.0)	17.6	1226 (100.0)	18.4
Age	0–2 years	354 (67.8)	18.4	862 (70.3)	17.8
	3–5 years	168 (32.2)	16.1	364 (29.7)	19.8
Reason for encounter^a^	Fever	167 (32.0)	12.0	464 (37.9)	17.2
	Cough	56 (10.7)	16.1	224 (18.3)	18.8
	Earache	41 (7.9)	9.8	77 (6.3)	19.5
	Vomiting	34 (6.5)	8.8	65 (5.3)	15.4
	Other RFE	285 (54.6)	22.8	595 (48.5)	19.5
Duration of symptoms	<5 h	166 (31.8)	13.3	273 (22.3)	13.2
	5–12 h	126 (24.1)	15.9	270 (22.0)	17.9
	12–24 h	86 (16.5)	15.1	290 (23.7)	15.5
	>24 h	130 (24.9)	26.9	387 (31.6)	24.6
	Missing	14 (2.7)	–	6 (0.5)	–
Time of contact	Weekdays 4–8 p.m.	110 (21.1)	24.6	216 (17.6)	22.7
	Weekday nights (8 pm - 8 am)	172 (33.0)	17.4	299 (24.4)	18.7
	Weekends and holidays	240 (46.0)	14.6	711 (58.0)	16.9
Geographical location	Urban	222 (42.5)	17.1	505 (41.2)	17.8
	Rural (<5000 inhab. Per postal code)	279 (53.5)	18.3	644 (52.5)	18.3
	Missing	21 (4.0)	–	77 (6.3)	–
Parent-assessed severity	Potentially severe	203 (38.9)	9.4	721 (58.8)	15.3
	Not severe	317 (60.7)	23.0	498 (40.6)	22.9
	Missing	2 (0.4)	–	7 (0.6)	–

^a^The five most frequent RFEs; numbers add up to more than the total as several RFEs were stated for some cases

According to the GPs, 17.6% of telephone consultations and 18.4% of face-to-face contacts were found to be more appropriate for the daytime GP (Table 2). Contacts at 4–8 p.m. on weekdays (24.6% of telephone consultations and 22.7% of face-to-face consultations) and contacts with symptom duration >24 h (26.9% and 24.6%, respectively) were most often assessed to be more relevant for daytime care than OOH-PC.

Table 3 depicts the parent-assessed problem severity in relation to the GP-assessed appropriateness of contacts for OOH-PC. For telephone consultations that were considered more appropriate for daytime care by the GPs, 79.4% of the consultations were assessed as non-severe by the parents. For face-to-face consultations, the corresponding figure was 50.7%. It should be noted that the agreement between GP-assessed appropriateness of contact and parent-assessed severity of health problem generally was low.

**Table 3:** Distribution of parent-assessed severity and GP-assessed appropriateness of OOH-PC contacts

		More appropriately targeted to the daytime GP			
	Parent-assessed severity	Yes (*n*=) (%)	No (*n*=) (%)	Don’t know (*n*=) (%)	All
Telephone consultations	Not severe	73 (79.4)	221 (56.5)	23 (59.0)	317 (60.7)
	Potentially severe	19 (20.6)	169 (43.2)	15 (38.5)	203 (38.9)
	Don’t know	0	1 (0.3)	1 (2.5)	2 (0.4)
	All	92 (100)	391 (100)	39 (100)	522 (100)
Face-to-face consultations	Not severe	114 (50.7)	346 (37.7)	68 (45.8)	498 (40.6)
	Potentially severe	110 (48.9)	566 (61.7)	45 (54.2)	721 (58.8)
	Don’t know	1 (0.44)	6 (0.6)	0	7 (0.6)
	All	225 (100)	918 (100)	83 (100)	1.226 (100)

Table 4 shows the 166 medically irrelevant contacts (assessed by the GPs to be inappropriately directed to OOH-PC and by the parents concern be non-severe problems). For telephone consultations, these contacts were statistically significantly more often seen on weekday evenings compared to weekday nights (adj. PR: 0.57 (95% CI: 0.34–0.95)) and weekends and holidays (adj. PR: 0.40 (95% CI: 0.23–0.68)). For face-to-face contacts, a similar trend (although not statistically significant) was seen. There was a tendency that longer symptom duration was associated with assessment as medically irrelevant for OOH-PC. Age of child and geographical location were not significantly associated with assessment as medically irrelevant for OOH-PC.

**Table 4:** Factors associated with medically irrelevant contacts (problems considered non-severe by parents and more appropriate for the daytime GP by triaging GPs)

Characteristics	Telephone consultations (*n* = 71^a^)			Face-to-face contacts (*n*=95^a^)		
	Prevalence	Adj. prevalence ratio^b^ (95% CI)	*p*-value	Prevalence	Adj. prevalence ratio^b^ (95% CI)	*p*-value
Age						
< 3 years	0.15	1 (reference)		0.09	1 (reference)	
3–5 years	0.13	0.99 (0.61–1.62)	0.982	0.11	1.41 (0.96–2.06)	0.077
Duration of symptoms						
< 5 h	0.13	1 (reference)		0.06	1 (reference)	
5–12 h	0.13	0.94 (0.50–1.75)	0.834	0.10	1.86 (0.95–3.62)	0.069
> 12–24 h	0.14	1.30 (0.67–2.49)	0.437	0.09	1.91 (0.99–3.69)	0.055
> 24 h	0.17	1.50 (0.86–2.61)	0.150	0.12	2.65 (1.45–4.86)	0.002
Time of contact						
4–8 pm on weekdays	0.22	1 (reference)		0.11	1 (reference)	
8 pm-8 am on weekdays	0.13	0.57 (0.34–0.95)	0.033	0.07	0.70 (0.40–1.22)	0.205
Weekends and holidays	0.11	0.40 (0.23–0.68)	0.001	0.10	0.69 (0.44–1.61)	0.112
Geographical location						
Urban	0.14	1 (reference)		0.09	1 (reference)	
Rural	0.15	1.11 (0.12–0.34)	0.639	0.09	1.11 (0.76–1.61)	0.588

^a^Two and 19 in telephone consultations and face-to-face consultations, respectively, excluded from analysis due to missing responses

^b^Adjusted for age groups, duration of symptoms and time of contact

## Discussion

### Main results

In 60% of telephone consultations and 40% of face-to-face consultations, parents considered the health problem as non-severe. In one fifth of these, the GPs also considered the problem as unappropriate for OOH-PC. For telephone consultations, we found that contacts during 4–8 p.m. on weekdays was significantly associated with assessment as medically irrelevant for OOH-PC, that is, considered to be more appropriately directed to daytime care by GPs *and* health problem was considered non-severe by parents. In face-to-face consultations, symptom duration of more than 12 h was significantly associated with medical irrelevance for OOH-PC.

### Strengths and limitations

Data originated from a large study on contacts to the OOH-PC service (LV-KOS), which ensured high statistical precision. Furthermore, the participating GPs and the random inclusion of contacts have been demonstrated to be highly representative of all contacts to OOH-PC [8]. As patient contacts were randomly recruited through electronic pop-up questionnaires and the participation rate among GPs was high, the risk of selection bias was limited. Respondents and non-respondents did not differ significantly on the included variables, except that the GPs rated the medical relevance for OOH-PC somewhat lower for non-respondents. As a larger share of the medically irrelevant contacts are likely to be perceived as non-severe by parents, we may have underestimated the number of contacts with low parent-assessed severity; if this is the case, our results represent conservative estimates of the investigated association.

The short time period between contacting OOH-PC and receiving the questionnaire reduced the risk of recall bias in the parents. Still, we cannot entirely rule out the possibility of recall bias as the parents assessed the severity of the health problem several days after the contact. Their child may have recovered, and this may have affected their response and their assessment of the treatment received. This might result in an underestimation of the severity in retrospect among parents and an overestimation of the proportion of contacts assessed as non-severe.

The share of contacts assessed as unappropriate for OOH-PC was similar for the two contact types. This may be surprising as the children seen in face-to-face consultations had already been triaged by a GP by telephone. However, the basis for the GP assessment of the medical appropriateness for OOH-PC varies by type of contact as the triaging GP only had information from the telephone interview and needed a safety margin. Therefore, some of these cases may have been referred to a face-to-face consultation even though they turned out to be medically irrelevant for OOH-PC. Moreover, according to the Danish clinical guidelines, all children below the age of six months with fever should be seen in a face-to-face consultation.

It would have been relevant to include RFEs in the regression analyses in a study focusing on medical relevance. However, due to the large diversity in RFEs, the statistical precision would be reduced by including RFEs in the multivariate analyses.

### Comparison with existing literature

Young children are more often sick than older children or adults [14, 15]. Therefore, this patient group is more frequently seen in OOH-PC; this group accounts for about one fifth of all calls [8, 16]. In accordance with other studies of children aged less than 5 years, we found fever, earache, coughing, breathing difficulties and vomiting the most common RFEs [12, 15, 17].

Our finding that many calls from parents to OOH-PC concern health problems in children which are often considered medically inappropriate for OOH-PC by the GPs is in line with results from other studies in similar settings [4–6, 18]. In a Norwegian survey on the urgency of calls to OOH-PC, 84.4% of calls regarding children below ten years of age were considered to concern non-urgent health problems [6]. Similarly, 44.9% of all contacts regarding children below two years of age and 55.8% contacts regarding children above two years of age to the emergency department of a paediatric department in Belgium were considered to concern non-urgent health problems [18]. In addition, a study showed that the rate of telephone consultations was almost four times as high for Danish children than for Dutch children [3]. The organisation of health care in general, including OOH primary care, is rather similar in the two countries [1]. Therefore, this difference could indicate that more contacts in Denmark concern health issues that are considered medically irrelevant for OOH-PC. Yet, previous studies indicate similar patterns of patient behaviour in the two countries [4].

We found several factors to be related to medical irrelevance for OOH-PC. The parents’ motives for contacting the OOH-PC have been investigated in several studies. Frequently mentioned motives were feelings of worry or fear, inadequateness to take care of their sick child, lack of control of their child’s condition and need for medical information or symptom relief [12, 15, 19]. To increase the quality of care and the efficiency in OOH-PC, it may be best for parents to wait and present their child’s health problem to the GP in the daytime. Moreover, the GP often knows the family beforehand and is more able to estimate the condition of the child using the already established relation with the parents. However, several studies indicate that this may not be possible in all cases as the psychosocial context of a child’s illness and the feeling of worry are likely to overrule other more rational reflections on the most relevant behaviour and the best use of resources [20–22].

Decisions on calling for medical advice are influenced by an array of various elements. Besides the actual medical problem, the parents’ knowledge and degree of feeling secure in managing their child’s illness are the main drivers for the decision-making [12]. Moreover, sociodemographic factors have been found to be associated with patient behaviour [23, 24]. These would have been relevant to include in the analyses of present study, but such information was not available. Future studies focusing on children in the OOH-PC should be designed to address the potential association between parent behaviour and sociodemographic factors.

### Generalisability and implications for research and practice

As parents frequently worry when their child is ill, it is relevant to aim directly at this issue in future studies to gain more knowledge and to identify potential ways of redirecting calls to daytime care. This may especially be relevant if calls are related to structural factors, such as perceived inaccessibility to own GP in the daytime, which has been shown to be associated with increased use of acute care services [25, 26]. The finding that most of the telephone consultations that were considered medically irrelevant for OOH-PC were seen during the first opening hours of the OOH-PC services on weekdays supports that this could be an important structural issue. As parents considered their child’s condition as non-severe in many cases, it would be relevant to examine more closely their motives for contacting the OOH-PC anyway. More insight in the reasons for contacting OOH-PC could provide input for better patient education and GP training. It would be particularly relevant to address the different elements that influence the motives for different help-seeking behaviour, such as individual sociodemographic factors and former experience with help-seeking. These components could be supplemented by exploring organizational factors related to the structure of the health care services, such as the accessibility to a GP within office hours.

## Conclusions

In this study of contacts concerning health issues in young children under six years of age to OOH-PC in Denmark, we found that fever, cough and ear pain were the most common RFEs. Parents assessed 60.7% of telephone consultations and 40.6% of face-to-face consultations to concern non-severe problems. GPs assessed that about 18% of contacts concerning children should have been directed to the daytime GP. Of these GP-assessed inappropriate contacts, parents considered the health problems as non-severe in 80% of the telephone consultations and in 50% of the face-to-face consultations. Contacts in the early evening on weekdays and contacts involving longer symptom duration (>24 h) were associated with medical irrelevance for OOH-PC. The results show varying perspectives between parents and GPs and confirm the challenge of defining the appropriate use of OOH-PC. Future studies focusing on parents’ motives and behaviour would be recommendable to establish more evidence on factors related to medical relevance of calls to OOH-PC in order to identify avoidable contacts.

## Abbreviations

CI: Confidence interval
GP: General practitioner
ICPC-2: International Classification of Primary Care, 2nd Edition (ICPC-2)
LV-KOS: Danish survey (and cohort) on contact and disease patterns in out-of-hours primary care
OOH-PC: Out-of-hours primary care
PR: Prevalence ratio
RFE: Reason for encounter
